# An Open-Source Horizontal Strabismus Simulator as an Evaluation Platform for Monocular Gaze Estimation Using Deep Learning Models

**DOI:** 10.3390/jemr19010020

**Published:** 2026-02-09

**Authors:** Shumpei Takinami, Yuka Morita, Jun Seita, Tetsuro Oshika

**Affiliations:** 1 Department of Ophthalmology, Faculty of Medicine, University of Tsukuba, Tsukuba 305-8575, Japan; s2130531@s.tsukuba.ac.jp (S.T.); jun.seita@riken.jp (J.S.); 2 Predictive Medicine Special Project, RIKEN Center for Integrative Medical Sciences, Tokyo 230-0045, Japan; 3 Division of Applied Mathematical Science, RIKEN Center for Interdisciplinary Theoretical and Mathematical Sciences, Saitama 351-0198, Japan

**Keywords:** strabismus, gaze estimation, deep learning, eye tracking, open-source hardware, simulator, monocular vision

## Abstract

Strabismus affects 2–4% of the global population, with horizontal cases accounting for more than 90%. Automated screening using monocular gaze estimation technology shows promise for early detection. However, existing models assume normal binocular vision, and their applicability to strabismus remains unvalidated due to the lack of evaluation platforms capable of reproducing disconjugate eye movements with known ground-truth angles. To address this gap, we developed an open-source, low-cost (approximately 200 USD) horizontal strabismus simulator. The simulator features two independently controllable artificial eyeballs mounted on a two-axis gimbal mechanism with servo motors and gyro sensors for real-time angle measurement. Mechanical accuracy achieved a mean absolute error of less than 0.1° across all axes, well below the clinical detection threshold of 1 prism diopter (≈0.57°). An evaluation of three representative AI models (Single Eye, GazeNet, and EyeNet) revealed estimation errors of 6.44–8.75°, substantially exceeding the clinical target of 2.8°. At this error level, small-angle strabismus (<15 prism diopters) would likely be missed, underscoring the need for strabismus-specific model development. Moreover, rapid accuracy degradation was observed beyond ±15° gaze angles. This platform establishes baseline performance metrics and provides a foundation for advancing gaze estimation technology for strabismus screening.

## 1. Introduction

Strabismus is a visual disorder that severely affects binocular visual function and represents a major ocular condition affecting 2–4% of the global population [1,2]. Patients face functional symptoms, such as diplopia and asthenopia, and psychosocial difficulties in self-image and social life [3,4]. Horizontal strabismus accounts for more than 90% of all strabismus cases [1,2]. Given this clinical prevalence, this study focuses on horizontal strabismus. To prevent these functional and psychosocial consequences, early detection and appropriate intervention are essential, particularly for preventing amblyopia and restoring binocular vision.

However, current ocular alignment examinations require specialized equipment and trained orthoptists and are primarily limited to ophthalmology clinics. Since only ophthalmology outpatient facilities have orthoptists who possess the specialized skills required for ocular alignment testing, opportunities to detect strabismus outside these settings are limited. While visual acuity testing is routinely performed in general screening examinations such as school health checkups and workplace health examinations, ocular alignment testing is rarely conducted. Consequently, mild-to-moderate strabismus is often overlooked and only discovered after symptoms have worsened or after others point out the ocular misalignment [5].

To overcome these barriers to early detection, various automated strabismus screening methods have been developed. Hartness et al. conducted a systematic review and meta-analysis. They demonstrated that a strong pooled correlation of 0.87 exists between automated strabismus evaluation devices and gold-standard clinical assessment [6]. Hirschberg test-based approaches, which estimate strabismus angle from corneal light reflex positions, have shown promising results, including an AI platform developed by Mao et al. [7]. However, these methods require strict control of lighting conditions and camera positioning. Eye-tracking-based methods have also been proposed, achieving high diagnostic accuracy through analysis of gaze deviation patterns [8]. These approaches require high-precision eye-tracking devices and mandatory calibration procedures, making implementation impossible without specialized equipment. Weber et al. demonstrated good agreement with conventional methods using infrared video goggles for automated Hess screen testing (ICC: 0.83 for horizontal and 0.76 for vertical) [9]. However, this approach is challenging to apply to children. Moreover, its widespread adoption is limited by low device availability and high implementation costs.

A promising alternative approach employs monocular gaze estimation technology based on deep learning. This method applies the principle of the cover test by independently estimating the gaze direction of each eye and calculating the strabismus angle from the difference [10]. Unlike Hirschberg-based methods, appearance-based gaze estimation can operate with standard RGB cameras without necessitating controlled lighting. VR-based studies [11] and research using custom devices [12] have demonstrated the feasibility of this approach. The practical realization of this technology could enable screening outside ophthalmology clinics using only smartphones or webcams, without requiring specialized equipment or expertise.

Despite this promise, substantial technical challenges remain in implementing strabismus screening using gaze estimation technology. Current state-of-the-art appearance-based methods employ convolutional neural networks (CNNs) trained on large-scale datasets. Representative examples include MPIIGaze and EVE, enabling gaze estimation in unconstrained real-world environments [13,14,15]. However, these datasets were collected exclusively from individuals with normal binocular vision, and the models implicitly assume that both eyes are aligned. Their performance under strabismic conditions, where the eyes exhibit disconjugate movements, remains entirely unknown. Before developing strabismus-specific gaze estimation models, it is essential to establish baseline performance metrics of existing models under such conditions.

Establishing such baseline metrics requires a systematic evaluation platform capable of reproducing disconjugate eye movements with known ground-truth angles. However, collecting large amounts of gaze data from actual strabismus patients is ethically and practically challenging [16,17]. Several synthetic eye image generators have been developed for gaze estimation research, but none adequately address this need. UnityEyes [18] generates monocular eye images using high-resolution 3D face scans and real-time rendering. However, they cannot produce binocular images and assume a kappa angle of zero. U2Eyes [19] extended this approach to binocular image generation. However, it assumes normal binocular vision and cannot model disconjugate eye movements. NVGaze [20] provides an anatomically informed synthetic dataset but does not model strabismus conditions. Moreover, developing such synthesis systems from scratch requires substantial resources, including 3D scanning equipment and multiple participants. Computational biomechanical models such as Orbit and SEE++ [21] simulate extraocular muscle mechanics for surgical planning. However, they generate numerical outputs rather than images suitable for training appearance-based models. GAN-based approaches such as StyleGAN2-ADA have been widely employed to generate realistic strabismus images for data augmentation [22]. However, they require labeled patient data for training and do not provide known ground-truth gaze angles for model evaluation.

Physical oculomotor simulators could serve as an alternative approach with known ground-truth eye position. However, existing systems do not support RGB image-based evaluation under strabismic conditions. Lotze et al. recently developed EyeRobot [23], a low-cost robotic simulator that emulates eccentric fixation and eye misalignment, with an estimated component cost of $200–500. However, this system was designed for validating infrared-based eye-tracking hardware using laser diodes for ground truth positioning, rather than for evaluating appearance-based deep learning models using RGB images. More sophisticated biomimetic systems with six degrees of freedom [24] or artificial muscle actuation [25] have been developed to examine oculomotor control. However, these are typically monocular and focused on replicating physiological dynamics rather than generating evaluation data for gaze estimation algorithms. Furthermore, many existing systems rely on proprietary software, raising concerns regarding research reproducibility and transparency [26,27,28]. No existing platform combines the three essential capabilities: reproducing strabismus conditions, generating RGB images, and providing known ground-truth gaze angles suitable for evaluating appearance-based gaze estimation models. This lack of evaluation infrastructure has been impeding the development of gaze estimation technology for strabismus screening.

To address this lack of evaluation infrastructure, we formulated the following three research questions:

(1) Can a low-cost, open-source physical simulator reproduce disconjugate eye movements? These movements are the defining characteristic of horizontal and vertical strabismus and must be reproduced with sufficient accuracy for AI model evaluation?
(2) How accurately can existing monocular gaze estimation models, trained on healthy subjects, estimate gaze direction under simulated strabismus conditions?
(3) Is the performance degradation at high gaze angles, previously reported in binocular gaze estimation, also observed in monocular gaze estimation?

Question (1) is a prerequisite for (2) and (3); without a strabismus simulator with accurate ground-truth angles, performance evaluation of AI models is impossible. In this study, we chose a physical simulator because existing digital simulators are unable to reproduce disconjugate eye movements. Moreover, modifying them would require substantial changes. However, a physical simulator can reproduce disconjugate eye movements simply by controlling each eyeball independently with servo motors, making implementation relatively straightforward. Additionally, physical simulators allow image capture with real cameras, providing lighting conditions and optical characteristics consistent with real-world environments. They offer more valid conditions for evaluating appearance-based deep learning models. Question (2) provides foundational data for assessing the applicability to strabismus screening. Question (3) analyzes the results of (2) by the angle range to clarify differences in detection difficulty between mild and severe strabismus.

To address these questions, we developed an independently controlled artificial eyeball system with real-time angle feedback using gyroscopic sensors. The manufacturing cost is approximately $200 USD. Additionally, the complete design information has been made publicly available, including 3D models, circuit diagrams, and software. Although a visual discrepancy (domain gap) between the artificial eyeballs and real human eyes, this limitation is addressed later. Using this platform, we evaluated three representative monocular gaze estimation models (Single Eye [29], GazeNet [30], and EyeNet [31]) and established baseline performance metrics under strabismic conditions.

A 3D face model with hollowed eye sockets was printed using the Adventure 5M Pro 3D printer, based on the Basel 3D Face Model [32]. The rotation mechanism consists of artificial eyeballs (Realistic eye; PARABOX, Kawasaki, Japan), 6-axis gyroscope sensors (MPU6050; InvenSense, San Jose, CA, USA), and servo motors for horizontal and vertical rotation (FS0307; FEETECH, Shenzhen, China), controlled by Arduino Nano microcontrollers (Arduino, Monza, Italy). Only horizontal rotation was evaluated in this study.

## 2. Materials and Methods

**Figure 1:**
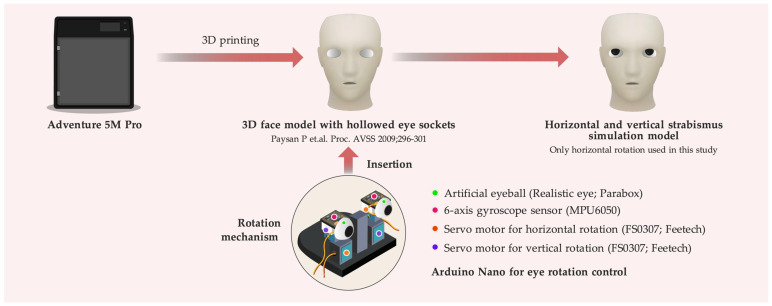
Schematic overview of the strabismus simulator development process [32].

The horizontal and vertical strabismus simulator developed in this study (Figure 1) was designed as a complete open-source hardware platform. All 3D model data (STL files), Arduino control software, and Processing source code (version 4.3.0, Processing Foundation, Boston, MA, USA) are publicly available on GitHub (https://github.com/namihira33/StrabismusSimulator, release v1.0.0; accessed on 6 February 2026) (see Supplementary Materials).

### 2.1. Hardware Design

**Figure 2:**
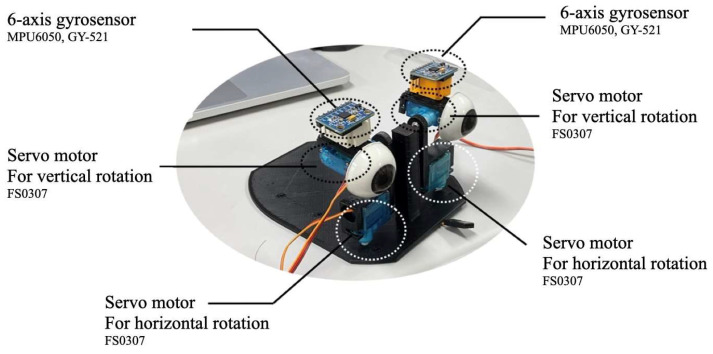
Hardware configuration of eyeball rotation system.

The strabismus simulator comprises two independently controllable artificial eyeballs mounted on a two-axis gimbal mechanism (Figure 2). Each eyeball can rotate horizontally and vertically using servo motors, providing real-time angle feedback through six-axis gyro sensors (MPU6050). Table 1 summarizes the main components required for system construction. These components were selected based on open-source hardware principles to maximize accessibility and reproducibility [26,27,28]. All parts are globally available and can be purchased for approximately 200 USD in total, enabling researchers worldwide to replicate the system.

The main mechanical components were designed in Fusion 360 (version 2605.0.97, Autodesk, San Francisco, CA, USA) and manufactured using a 3D printer. For the face model, the bilateral ocular regions were hollowed out from the Basel 3D Face Model [32], and the interior was made hollow to reduce weight and printing time. Motor holders for horizontal and vertical rotations were designed to precisely fit the dimensions of the FS0307 servo motors. The two-axis gimbal design achieved a range of motion of ±30° in both horizontal and vertical directions. All the 3D models were printed with a PLA filament (1.75 mm) using a FlashForge Adventure 5M Pro printer (FlashForge, Jinhua, China).

**Figure 3:**
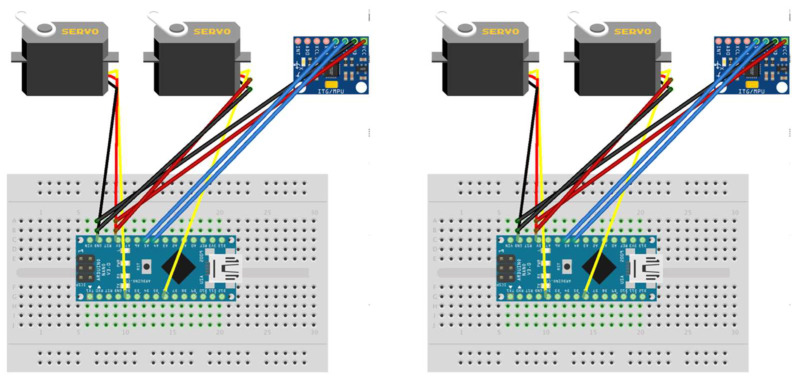
Circuit configuration of motor control system.

The control system was configured using two independent Arduino Nano microcontrollers, controlling each eyeball individually (Figure 3). An MPU6050 sensor was connected to each microcontroller, acquiring six-axis data (three-axis acceleration and three-axis gyro). The acquired sensor data were processed by a Madgwick filter, achieving stable pose estimation with minimal drift.

The system features two independently controllable artificial eyeballs, each equipped with horizontal and vertical servo motors (FS0307) and 6-axis gyro sensors for real-time angle measurement. The gyro sensor used in this system is the MPU6050, a Micro Electro Mechanical Systems (MEMS)-based inertial measurement unit, mounted on a GY-521 breakout board that provides an I2C interface for communication with the Arduino Nano microcontrollers. Control is implemented with Arduino Nano microcontrollers connected via breadboard.

Independent control circuits for the left eye (left) and right eye (right). Each system consists of an Arduino Nano microcontroller, MPU6050 gyro sensor (I2C connection), and two FS0307 servo motors (for horizontal and vertical control) In Figure 3, colored lines represent wiring connections: red indicates power supply (5V), black indicates ground (GND), and other colors represent signal lines for I2C communication and servo control. Dots on the breadboard indicate electrical connection points.

For binocular motion control, to simulate strabismus conditions, the left eye was set to an initial position of 60° and the right eye to 90°, designed to perform random movements within a range of ±30° each. Using the same random seed for both eyes ensures complete reproducibility between experiments while still allowing independent movements. The total manufacturing cost of the system is significantly low at approximately 200 USD, enabling seamless adoption even by research institutions with limited budgets.

### 2.2. System Validation

**Figure 4:**
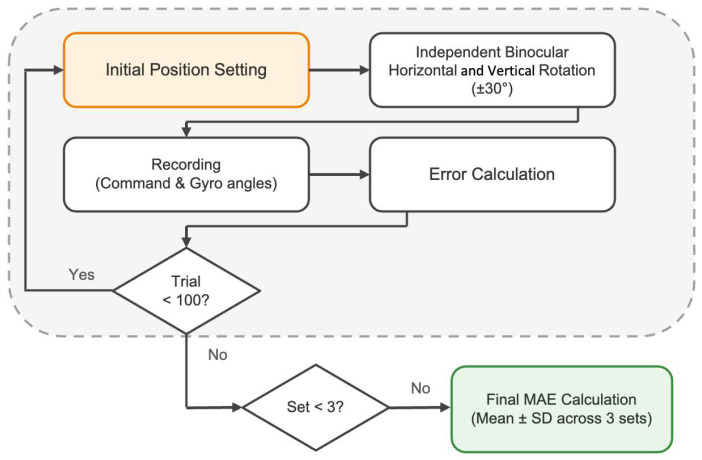
Flowchart of the system validation protocol.

To evaluate the angular accuracy of the system, we implemented standardized measurement protocols and data analysis methods. Each simulator eye was independently and randomly rotated within a range of ±30° horizontally and vertically, and 100 trials were performed per set (Figure 4). Pose angles were calculated by integrating three-axis angular velocity and three-axis acceleration data using the Madgwick filter, minimizing the effects of gyro drift and acceleration noise. The filter parameters are specified in the Arduino source code available in the GitHub repository. System accuracy was quantitatively evaluated by calculating the Mean Absolute Error (MAE) and standard deviation from a comparison of the collected servo command angles and gyro-measured angles.

Each set consisted of 100 trials of independent binocular rotation (±30° horizontal and vertical), and the final mean absolute error was calculated as the mean ± SD across three sets.

The gaze angle is represented as a two-dimensional vector $\varphi =\left(\varphi_{x},\varphi_{y}\right)$, where $\varphi_{x}$ denotes the horizontal component (yaw) and $\varphi_{y}$ denotes the vertical component (pitch), both expressed in degrees. The coordinate system defines the frontal gaze direction as the origin (0°, 0°), with positive $\varphi_{x}$ indicating rightward (temporal) rotation and positive $\varphi_{y}$ indicating upward rotation.

The mechanical accuracy was evaluated using the MAE:

(1) $$\begin{array}{c} MAE\;=\frac{1}{n}\sum_{i=1}^{n}\left|\varphi_{cmd,i}-\;\varphi_{gyro,i}\right| \end{array}$$

where $\varphi_{cmd,i}$ is the commanded rotation angle sent to the servo motor for the i-th trial, $\varphi_{gyro,i}$ is the corresponding angle measured by the MPU6050 gyroscope sensor, and n is the total number of trials. This metric was calculated independently for each axis ($\varphi_{x}$ and $\varphi_{y}$) of both eyes. To ensure reliability, three sets of 100 trials were performed, and the final MAE was calculated as the mean ± SD across the three sets.

### 2.3. Data Collection for AI Model Evaluation

**Figure 5:**
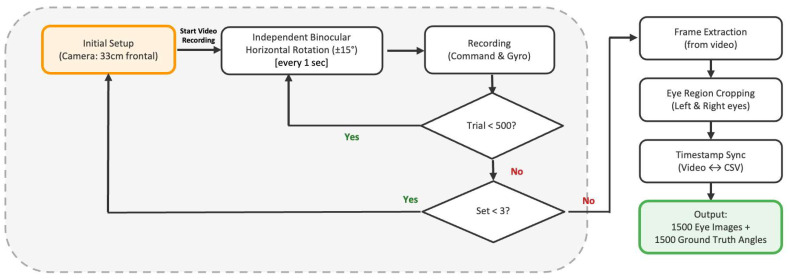
Flowchart of the data collection protocol for AI model evaluation.

Data for AI model evaluation was collected independently for left and right eyes under each condition (Figure 5). To simulate clinically relevant strabismus conditions within the range of ±15°, both artificial eyeballs were independently rotated within the specified angle ranges from the forward-facing position. The simulator was programmed to change the rotation angles of both eyeballs every second. This continuous motion was recorded as video footage using a Samsung Galaxy Z Flip 3 (Samsung Electronics, Suwon, South Korea) smartphone positioned 33 cm away from the simulator, facing the artificial eyeballs frontally. The video resolution was 1920 × 1480 pixels with auto exposure settings.

Each set consisted of 500 trials independent of binocular horizontal rotation (±15°) recorded every 1 s, yielding 1500 eye images with synchronized ground truth angles across three sets.

The recorded data were processed to generate paired eye images and ground truth angles. Individual frames were extracted from the recorded video, and eye regions were subsequently cropped from these frames for use as model input. Eye regions were detected using MediaPipe Iris (version 0.10.2, Google, Mountain View, CA, USA) running on Python (version 3.9.0, Python Software Foundation, Wilmington, DE, USA), cropped to 60 × 36 pixels, and preprocessed by converting to grayscale and applying histogram equalization before input to the models. Ground truth gaze angles were simultaneously recorded using the 6-axis gyro sensors (MPU6050) mounted on each artificial eyeball. Synchronization was achieved through Processing software, which provided unified control over the timing of rotation commands sent to the Arduino and angle measurements. The Processing source code is publicly available in the GitHub repository. The simulator was designed to receive rotation commands at 1 s intervals and acquire angle data after rotation completion. This ensures temporal correspondence between video frames and gyro measurements on a per-rotation-cycle basis. The measured data were recorded in CSV format with timestamps. This synchronization allowed precise correlation between the eye images and actual rotation angles. Additionally, to examine the effect of gaze angle range on estimation accuracy, data were collected at six range levels (±5° to ±30° in 5° increments) following the same protocol.

### 2.4. Evaluation of AI Models for Monocular Gaze Estimation

As an initial step toward clinical application, we conducted a fundamental performance evaluation of representative AI models for monocular gaze estimation using a simulator developed in this study. Three models were selected as baselines for evaluation: Single Eye (four-layer CNN structure), GazeNet (VGG16-based) pre-trained on the MPIIGaze dataset, and EyeNet (ResNet18-based) pre-trained on the EVE dataset. These three models were selected because their training datasets and model architectures are publicly available, ensuring reproducibility. All models were implemented and evaluated using PyTorch (version 1.9.1, Meta AI, New York, NY, USA). They also represent a progression in architectural complexity, from a simple four-layer CNN (Single Eye) to VGG16-based (GazeNet) and ResNet18-based (EyeNet). This section enables the assessment of whether increased model complexity improves performance under strabismus conditions. All three models share a common approach consisting of feature extraction layers followed by fully connected layers to estimate gaze direction from monocular eye images. Cheng et al. demonstrated that this CNN-based appearance feature extraction approach is widely adopted as a standard method in current monocular gaze estimation research [15]. The estimated angle error (angular difference between model estimates and gyro sensor measurements) was used as the performance metric. The clinical target accuracy was set at 2.8°, based on the inter-examiner variability of the alternate prism cover test (APCT). This variability has been reported to be approximately 7 prism diopters (PD) or 4.01° (1 PD ≈ arctan (1/100) ≈ 0.57°) [33]. According to measurement error theory, averaging multiple independent measurements reduces the standard error in proportion to the square root of the number of measurements [34]. This method estimates the gaze direction of each eye independently and calculates the strabismus angle from the difference between them. Moreover, it assumes that the estimation errors of each measurement are independent, corresponding to the average of two measurements. Thus, the target precision becomes approximately 4.01°/√2 ≈ 2.8°.

In this study, we examined the impact of gaze angle range on estimation accuracy by systematically varying the range across six levels (±5° to ±30° in 5° increments). We aimed to determine the minimum detectable angle for clinical applicability. We selected EyeNet because it is the most recent model among the three baselines. Data collection followed the same protocol described in Section 2.3.

Gaze estimation accuracy was evaluated using the MAE between the estimated and ground truth angles:

(2) $$\begin{array}{c} {MAE}_{gaze}=\frac{1}{n}\sum_{i=1}^{n}\left|{\hat{\varphi }}_{x,i}-\;\varphi_{x,i}\right| \end{array}$$

where ${\hat{\varphi }}_{x,i}$ is the horizontal gaze angle estimated by the AI model for the i-th sample, $\varphi_{x,i}$ is the ground truth horizontal angle measured by the gyroscope sensor, and n is the total number of samples. Only the horizontal component $\varphi_{x}$ was evaluated in this study, as the simulator was configured for horizontal rotation only during AI model evaluation.

## 3. Results

### 3.1. System Performance

The developed simulator demonstrated excellent mechanical accuracy and linear response characteristics across all four axes. The correlation analysis between servo command angles and gyro-measured angles recorded a perfect correlation coefficient of 1.000 (*p* < 0.001) and a coefficient of determination R^2^ = 1.000 for all axes, confirming extremely high linearity. The regression line slopes were 0.983 for right eye horizontal, 0.991 for right eye vertical, 0.991 for left eye horizontal, and 0.998 for left eye vertical.

**Figure 6:**
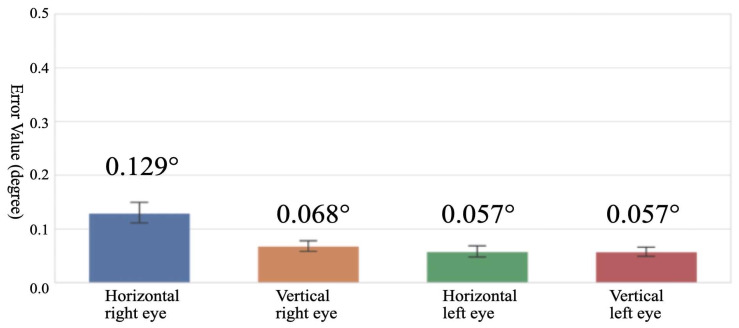
Mean absolute error between measured values and command angles.

As shown in Figure 6, the absolute angle error evaluation recorded 0.129 ± 0.099° for right-eye horizontal, 0.068 ± 0.052° for right-eye vertical, 0.057 ± 0.054° for left-eye horizontal, and 0.057 ± 0.043° for left-eye vertical, achieving high accuracy at the 0.1° level for all axes. This accuracy is significantly below the minimum detection unit of 1 prism diopter used in clinical diagnosis.

The bar chart shows the mean absolute error between the servo command angles and the gyro sensor-measured angles for four axes: horizontal/vertical movements of the right and left eyes. Error bars represent standard deviations.

### 3.2. AI Model Validation

**Figure 7:**
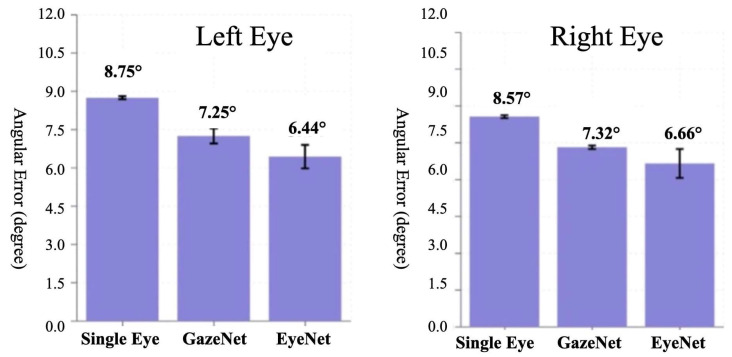
Mean angle errors of each model.

Initial verification using three representative monocular gaze estimation models revealed fundamental performance characteristics in the strabismus simulator environment. As shown in Figure 7, in terms of model performance, ResNet18-based EyeNet exhibited the best results, recording estimation angle errors of 6.44° and 6.66° for the left and right eyes, respectively. VGG16-based GazeNet showed errors of 7.25° and 7.32° for the left and right eyes, respectively. However, the four-layer CNN Single Eye showed errors of 8.75° and 8.57°, confirming that the deep learning architecture contributes to performance improvement.

The bar charts display the estimation errors for the Left Eye (left panel) and Right Eye (right panel) using Single Eye, GazeNet, and EyeNet models. The *y*-axis represents the angular error in degrees. Error bars indicate standard errors.

**Figure 8:**
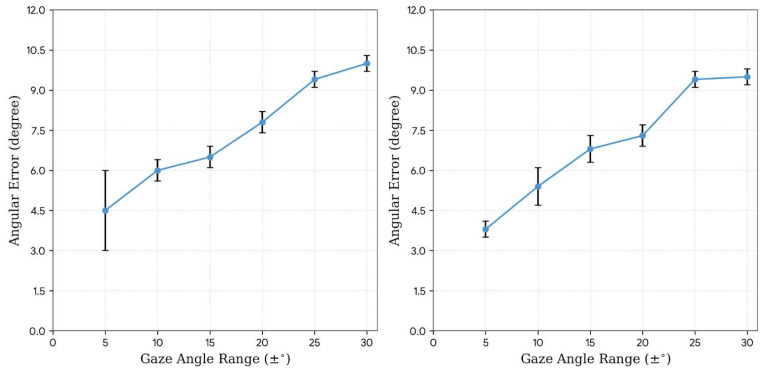
Gaze angle error and error range of EyeNet.

As shown in Figure 8, estimation accuracy decreased progressively as the gaze angle range increased. Angular error remained relatively stable within ±15° but showed marked degradation beyond this range, accompanied by increased variance. These results suggest that appearance-based gaze estimation may be more reliable for detecting small-to-moderate angle strabismus, while large-angle deviations present greater challenges for existing architectures.

The plots show the mean angular error (*y*-axis) as a function of the gaze angle range (*x*-axis), varying from ±5° to ±30°. The left and right panels correspond to the results for the left and right eyes, respectively. Error bars indicate standard errors.

## 4. Discussion

This study addressed three research questions regarding the evaluation of gaze estimation AI models under strabismus conditions. Regarding Question (1) (simulator accuracy), the developed physical simulator achieved mechanical accuracy with an MAE below 0.2° in both horizontal and vertical directions. This accuracy is below 1 PD (approximately 0.57°), the minimum measurement unit used clinically. Therefore, the simulator functions as a ground truth data foundation with sufficient reliability for AI model evaluation. Furthermore, the ability to independently reproduce disconjugate eye movements, a primary characteristic of strabismus, in both eyes was demonstrated. Regarding Question (2) (model performance under simulated strabismus conditions), all three monocular gaze estimation models (Single Eye, GazeNet, and EyeNet) exhibited estimation errors ranging from 6.44° to 8.75°, failing to achieve the clinical target accuracy of 2.8°. These results indicate that existing models developed under the assumption of normal binocular vision cannot be directly applied to monocular gaze estimation for strabismus screening. Regarding Question (3) (the relationship between gaze angle range and estimation accuracy), a trend of decreasing accuracy with expanding gaze angle range was confirmed. The MAE was approximately 4.5° in the ±5° range, increasing to approximately 10° in the ±30° range. These findings demonstrate that the performance degradation at higher gaze angles reported in binocular gaze estimation is similarly observed in monocular gaze estimation.

### 4.1. Clinical Interpretation

In this study, we considered the clinical implications of the estimation errors of 6.44° to 6.66° (EyeNet). Strabismus severity is generally classified using prism diopters (PD): small-angle deviations (<15 PD, approximately <8°), moderate deviations (15–30 PD, approximately 8–17°), and large-angle deviations (≥30 PD, approximately ≥17°) [35]. Given the current estimation errors of 6.44° to 6.66°, reliable detection of small-angle strabismus (<15 PD) is challenging. However, moderate or greater deviations (≥15 PD, approximately ≥8°) fall within the detectable range even when accounting for estimation errors. Therefore, applying current models to clinical screening could pose a risk of missing small-angle strabismus, where early detection is most critical for preventing amblyopia. This highlights the need to develop gaze estimation models specially designed for strabismus screening.

The analysis by gaze angle range exhibited the highest accuracy (MAE approximately 4.5°) within the ±5° range, which is still below the clinical target of 2.8°. In contrast, the MAE increased to approximately 10° in the ±30° range, with substantially greater variability in estimation errors. Therefore, different approaches are required for mild versus severe strabismus. For severe strabismus, accurately quantifying eye gaze misalignment using gaze estimation remains challenging. Moreover, it necessitates the consideration of alternative strategies, such as approaches specialized for detection (presence or absence) or combination with other examination methods. For mild strabismus screening, fundamental improvements to current architectures are also essential. These findings underscore the importance of a strategic approach designed according to target strabismus severity in the future development of monocular gaze estimation models for strabismus screening. They also provide essential baseline data for determining subsequent research directions.

### 4.2. Comparison with Existing Evaluation Platforms

A comparison of this simulator with existing gaze estimation evaluation systems revealed several important characteristics. Digital simulators such as UnityEyes [18] and U2Eyes [19] are effective for large-scale synthetic data generation. However, they cannot fully replicate the optical properties of physical eyeballs and lack functionality to reproduce disconjugate eye movements. EyeRobot [23] is a low-cost ($200–500) physical simulator designed for infrared-based eye tracking verification. However, it is unsuitable for evaluating appearance-based deep learning models using RGB images. This simulator is the first platform to combine three essential functions, reproduction of strabismus conditions, RGB image generation, and providing known ground-truth gaze angles, at a low cost of approximately $200.

Regarding AI evaluation results, compared to reported accuracies of Single Eye (approximately 6°) [29] and GazeNet (approximately 5°) [30] on the MPIIGaze dataset, the error values observed in this study (8.57–8.75° and 7.25–7.32°, respectively) were larger. This performance degradation is attributable to two factors: First, domain shift between training data (real eyes of healthy individuals) and evaluation environment (artificial eye simulator). Artificial eyes lack fine features in real eyes, such as iris patterns, dynamic pupil changes, and scleral vascular patterns. This impaired feature extraction is dependent on these characteristics. Second, the strabismus condition. Existing models were trained assuming normal binocular vision and may perform poorly under the unfamiliar condition of disconjugate eye movements. The experimental design of this study did not allow separate quantification of these two factors. Future studies must use data from actual strabismus patients to separately analyze the effects of domain shift due to artificial eyes and those attributable to strabismus conditions.

### 4.3. Scientific Contributions

The scientific contributions of this study can be summarized in three points. First, we provided the first systematic platform for evaluating gaze estimation models under strabismus conditions. This platform is capable of reproducing disconjugate eye movements with known ground truth angles. Moreover, it enables quantitative measurement of model performance under strabismus conditions, a capability that was previously unavailable. Second, we quantified for the first time the performance limitations of existing models trained on healthy individual data under strabismus conditions. All three representative models failed to achieve clinical target accuracy, highlighting the need to develop dedicated models for monocular gaze estimation for strabismus screening. Third, we systematically analyzed the relationship between gaze angle range and estimation accuracy in monocular gaze estimation for the first time, revealing differences in detection difficulty between mild and severe strabismus. These findings provide baseline data for setting target strabismus angle ranges in future model development.

### 4.4. Future Direction

Based on the issues identified in this study, the following directions are considered. First, this study is positioned as preliminary research enabling clinical validation. The demonstration of existing model limitations through simulator evaluation establishes both the scientific rationale and ethical justification for subsequent validation studies with actual strabismus patients. Future work must verify the correlation between simulator evaluation results and actual patient measurements to confirm the clinical validity of this platform. Second, the construction of strabismus-specific datasets is crucial. Utilizing this simulator to systematically generate synthetic data that reproduces various strabismus patterns (exotropia, esotropia, vertical strabismus, etc.) and combining these with actual patient data allows the creation of sufficient training datasets. This approach also alleviates the ethical and practical constraints associated with data collection. Third, the application of domain adaptation techniques is promising. Applying transfer learning or adversarial learning methods to reduce distributional differences between the training domain (real eyes of healthy individuals) and the target domain (strabismus patients) is expected to improve the applicability of existing models to strabismus conditions. Domain shift between artificial and real eyes may also be mitigated using similar approaches. Fourth, while this simulator supports both horizontal and vertical eye movements, this study limited evaluation to horizontal strabismus, which yielded the highest clinical frequency (>90%). Future work must expand the evaluation scope to include vertical and combined strabismus.

### 4.5. Limitations

This study has several limitations. First, a domain gap exists between artificial and real eyes. Artificial eyes do not fully replicate human iris patterns, dynamic pupil diameter changes, scleral vascular patterns, or corneal optical properties. This domain shift affects AI evaluation results, and different outcomes may be obtained when evaluating actual strabismus patients. Part of the performance degradation observed in this study is attributable to this domain shift and must be interpreted separately from performance degradation due to strabismus conditions. Second, this study did not conduct clinical validation. The correlation between simulator measurements and actual patient measurements has not been established and requires future validation studies. However, this study is positioned as preliminary research enabling such clinical validation. Third, this initial validation did not systematically vary environmental factors such as lighting conditions and camera position. Evaluation under diverse environmental conditions is crucial for clinical application.

## 5. Conclusions

This study addressed three research questions regarding the evaluation of gaze estimation AI models under strabismus conditions and obtained the following findings. Regarding Question (1), the developed low-cost (approximately US$200) open-source physical simulator achieved mechanical accuracy with an MAE below 0.2° in both horizontal and vertical directions. This accuracy is below 1 PD, the minimum measurement unit used clinically. Therefore, the simulator serves as a ground-truth data foundation with sufficient reliability for AI model evaluation. Furthermore, we demonstrated the ability to independently reproduce disconjugate eye movements, a primary characteristic of strabismus in both eyes. Regarding Question (2), three representative monocular gaze estimation models trained on healthy individual data exhibited estimation errors ranging from 6.44° to 8.75°, failing to achieve the clinical target accuracy of 2.8°. At this error level, detection of microtropia and mild strabismus is challenging. This indicated a high risk of missing mild strabismus, where early detection is critical. Regarding Question (3), a trend of decreasing accuracy with expanding gaze angle range was observable, with MAEs of 4.5° in the ±5° range and 10° in the ±30° range. These results suggest that different approaches are essential for mild versus severe strabismus, providing essential baseline data for future model development.

The scientific contributions of this study are summarized in three points: provision of a model evaluation platform under strabismus conditions, the first quantification of existing model performance limitations, and systematic analysis of the relationship between gaze angle range and estimation accuracy. This study is positioned as preliminary research before clinical validation. The baseline metrics provide quantitative standards for future development of models specialized for monocular gaze estimation for strabismus screening and detailed analysis of domain shift. By making the design fully open-source, we established an environment enabling participation in strabismus research even for institutions with budget constraints.

## Figures and Tables

**Table 1:** Parts list for strabismus simulator.

Component	Product	Quantity
Microcontroller	Arduino nano	2
6-axis gyro sensor	MPU6050	2
Servo motor	FS0307	4
3D-printed material	PLA filament	300 g
Artificial eyeball	Realistic eye	2

## Data Availability

The original contributions presented in this study are included in the article. The design files and source code are publicly available at: https://github.com/namihira33/StrabismusSimulator (release v1.0.0; accessed on 6 February 2026).

## Supplementary Materials

The following supporting information can be downloaded at: https://github.com/namihira33/StrabismusSimulator (release v1.0.0; accessed on 6 February 2026). Data S1: 3D model files (STL); Data S2: Arduino control software; Data S3: Processing source code.
